# [68Ga]Pentixafor-PET/MRI for the detection of Chemokine receptor 4 expression in atherosclerotic plaques

**DOI:** 10.1007/s00259-017-3831-0

**Published:** 2017-09-21

**Authors:** Xiang Li, Daniel Heber, Tatjana Leike, Dietrich Beitzke, Xia Lu, Xiaoli Zhang, Yongxiang Wei, Markus Mitterhauser, Wolfgang Wadsak, Saskia Kropf, Hans J. Wester, Christian Loewe, Marcus Hacker, Alexander R. Haug

**Affiliations:** 1 Division of Nuclear Medicine, Department of Biomedical Imaging and Image-guided Therapy, Medical University of Vienna, Währinger Gürtel 18-20, 1090 Vienna, Austria; 2 Division of Cardiovascular and Interventional Radiology, Department of Biomedical Imaging and Image-guided Therapy, Medical University of Vienna, Vienna, Austria; 3 Department of Nuclear Medicine, Beijing Anzhen Hospital, Capital Medical University, Beijing, China; 4 Ludwig Boltzmann Institute Applied Diagnostics, Vienna, Austria; 5 Center for Biomarker Research in Medicine, CBmed, Graz, Austria; 6 Scintomics GmbH, Fürstenfeldbruck, Germany; 7 Department of Radiopharmaceutical Chemistry, Technische Universität München, Garching, Germany

**Keywords:** Chemokine receptor type 4, [^68^Ga]Pentixafor, Atherosclerosis, PET/MRI

## Abstract

**Purpose:**

The expression of chemokine receptor type 4 (CXCR4) was found co-localized with macrophages on the atherosclerotic vessel wall and participated in the initial emigration of leukocytes. Gallium-68 [^68^Ga]Pentixafor has recently been introduced for the imaging of atherosclerosis by targeting CXCR4. We sought to evaluate human atherosclerotic lesions using [^68^Ga]Pentixafor PET/MRI.

**Methods:**

Thirty-eight oncology patients underwent [^68^Ga]Pentixafor PET/MR imaging at baseline. Maximum standardized uptake values (SUV_max_) were derived from hot lesions in seven arterial segments and target-to-blood ratios (TBR) were calculated. ANOVA post-hoc and paired t test were performed for statistical comparison, Spearman’s correlation coefficient between uptake ratios and cardiovascular risk factors were assessed. The reproducibility of [^68^Ga]Pentixafor PET/MRI was assessed in seven patients with a follow-up exanimation by Pearson’s regression and Bland–Altman plots analysis.

**Results:**

Thirty-four of 38 patients showed 611 focal [^68^Ga]Pentixafor uptake that followed the contours of the large arteries. Both prevalence and mean TBR_max_ were highest in the descending aorta. There were significantly higher TBR values found in men (1.9 ± 0.3) as compared to women (1.7 ± 0.2; *p* < 0.05). Patients with mean TBR_max_ > 1.7 showed a significantly higher incidence of diabetes, hypertension hypercholesterolemia and history of cardiovascular disease than patients with mean TBR_max_ ≤ 1.7. [^68^Ga]Pentixafor uptake showed a good reproducibility (*r* = 0.6, *p* < 0.01), and there was no difference between the mean TBR_max_ values of plaque lesions (TBR_baseline_1.8 ± 0.3 vs TBR_follow-up_1.8 ± 0.3) (*p* = 0.9).

**Conclusion:**

Patients with high arterial uptake showed increased incidence of cardiovascular risk factors, suggesting a potential role of [^68^Ga]Pentixafor in characterization of atherosclerosis.

## Introduction

Leukocyte recruitment to the injured endothelium and subsequent plaque formation is an important step throughout the progression of atherosclerosis, which is mainly regulated by chemokines [1, 2]. Specifically, the C-X-C receptor 4 (CXCR4) and its endogenous ligand, the C-X-C motif chemokine ligand (CXCL12) have been implicated in the hematopoiesis in bone marrow through modulation of the progenitor cells that home to the bone marrow and move into peripheral inflammatory tissues [3], including apoptosis, and proinflammatory progression in atherosclerotic plaques [4]. In addition, the combination of CXCR4 and its alternative ligand macrophage inhibitory factor (MIF) plays an important role in the recruitment of leukocytes into the intima of vessel walls after endothelial injury, a process which is crucial for the progression of atherosclerosis [5].

In pioneering studies, the CXCR4/CXCL12 axis has been found to be essential in various autoimmune diseases, such as rheumatoid arthritis [6], systemic lupus erythematosus [7], and autoimmune disorders of the nervous system [8]. CXCR4 also accumulates progressively during atherosclerosis progression by modulating neutrophil migration, as well as specifically co-localizing with macrophage infiltration [9]. In pathophysiology studies, CXCR4 was proven to be intensively expressed on hematopoietic progenitor cells, immature thymocytes, naive B and T lymphocytes [10], monocytes/marcophage [4, 11], and dendritic cells [12].

The newly developed, integrated PET/MRI scanners were introduced to cardiovascular imaging with high expectations, especially for the characterization of the internal structure of atherosclerotic artery walls due to the excellent soft tissue contrast of MRI [13]. Comprehensive characterization of atherosclerosis might benefit from hybrid PET/MRI to delineate the vessel wall and the activity of atherosclerotic lesions.

The novel PET tracer, [^68^Ga]Pentixafor [14] with high affinity to CXCR4, has recently been introduced for the imaging of several different hematologic and other neoplasms including leukemia, lymphoma, multiple myeloma, adrenocortical carcinoma or small cell lung cancer [15–19], and also in other solid tumors and disease conditions, such as splenosis, stroke, atherosclerosis, and myocardial infarction in humans and in animals [9, 20, 21]. In the present studies, we sought to evaluate the reproducibility of [^68^Ga]Pentixafor uptake quantification of atherosclerotic lesions with PET/MRI. Furthermore, we sought to evaluate the relation between CXCR4 expression and the cardiovascular risk profile of the patients.

## Methods and materials

### Patient population

Thirty-eight oncological patients (28 lymphoma and 10 pancreatic cancer patients) with detected arterial focal uptake were assessed in this retrospective analysis of ongoing prospective trials, where all patients provided written informed consent. Four lymphoma patients had to be excluded due to difficulties in separating vascular [^68^Ga]Pentixafor uptake from adjacent lymph nodes. All patients underwent [^68^Ga]Pentixafor PET/MR imaging for staging or restaging purposes. Relevant baseline characteristics of the patients (*n* = 34) are reported in Table 1. Seven lymphoma patients also underwent follow-up [^68^Ga]Pentixafor PET/MR scans 111 ± 38 days after baseline. These patients were considered for reproducibility assessment. The clinical institutional review board approved this study.

**Table 1:** Patient characteristics

Baseline patients characteristics (*n* = 34)	All patients
Age, mean ± SD	67 ± 11
Men, n (%)	17(50%)
Lymphoma, n (%)	24(71%)
Pancreatic cancer, n (%)	10(29%)
Body-mass index(kg/m2), mean ± SD	26 ± 4
Risk factors, n (%)	
Hypertension	15(44%)
Diabetes type II	6(18%)
Hypercholesterolemia	9(26%)
Smoking	7(21%)
History of cardiovascular diseases	9(26%)
Family history of cardiovascular disease	4(12%)
Medication for cardiovascular diseases, n (%)	
Statin therapy	9(26%)
Angiotensin-converting-enzyme (ACE) inhibitor	7(21%)
Beta Blocker	9(26%)
Calcium antagonists	3(9%)
Diuretic therapy	4(12%)
Aspirin	3(9%)
Treatment between baseline and follow-up (n = 7), n (%)	
Chemotherapy	6 (86%)
Radiotherapy	2 (29%)
Chemo- and radiotherapy	2 (29%)

### Synthesis of [^68^Ga]Pentixafor

[^68^Ga]Pentixafor production was carried out as already described [22, 23] in a fully automated manner by using a Scintomics GRP module. Briefly, the eluate of a ^68^Ge/ ^68^Ga generator was concentrated using a cation exchange cartridge, mixed with a solution of Pentixafor and buffered with HEPES. After conversion at 125 °C (pH ~ 5) for 6 min, [^68^Ga]Pentixafor was purified using a C18 solid phase extraction cartridge (Waters SepPak light), eluted with ethanol/water and formulated in 14 mL of PBS buffer (pH 7.4). Full radiopharmaceutical quality control of [^68^Ga]Pentixafor was carried out according to the methods described in the European Pharmacopeia.

### PET/MRI

Patients after injection of 165 ± 29 MBq [range: 78 to 229 MBq] [^68^Ga]Pentixafor underwent PET/MR imaging (Biograph mMR, Siemens Healthcare GmbH, Erlangen, Germany) to assess CXCR4 expression. PET/MR images were acquired in five bed positions with a 5 min per bed position. The MR imaging component was performed with an integrated radiofrequency coil and a multi-station protocol, with a slice thickness of 2 mm. Time of flight (TOF) magnetic resonance angiography (MRA) of both carotid arteries was performed for delineation of carotid stenoses. Attenuation correction was performed using the implemented standard four-compartment model attenuation map calculated from a Dixon-based VIBE (volumetric interpolated breath-hold examination) sequence. A 3-D ordinary Poisson ordered subsets expectation maximization (OP-OSEM) algorithm, with PSF correction and three iterations and 21 subsets, was used for reconstruction. The image matrix size was 172 × 172 (pixel size 4.2 mm). The images were smoothed with a 3-mm full-width at half-maximum (FWHM) Gaussian filter.

### Image analysis

Reconstructed PET/MR images were analyzed as previously described [13]. All axial PET image slices were inspected visually along seven arterial segments including left and right carotid artery, aortic arch, ascending and descending aorta, and abdominal aorta. Subsequently, maximum arterial uptake was derived from 3-D-volumes of interest (VOI), which were drawn at the visualized arterial lesions (TOF MRA positive lesions with focal uptake in the carotid arteries; focal uptake in the aortic lesions). As a reference, SUV_bloodpool_ was calculated as the mean SUV of three ROIs (diameter of 10 mm) within the lumen of the vena cava. TBRs were calculated by respective SUVmax values corrected for background blood-pool activity [24, 25]. Mean TBRmax was defined as the average value of TBRmax derived from VOIs.

$$ TBRmax= SUVmax\div SUVmean\left( blood pool\right) $$

### Immunohistochemistry

To detect CXCR4 expression, immunohistochemistry was performed on cryosections from carotid endarterectomy specimens (six carotid plaque lesions from three patients provided by Dr. Jie Du, Beijing Institute of Heart, Lung and Blood Vessel Diseases, Beijing Anzhen Hospital, China). Utilization of human plaque tissues was approved by the ethics committee of the medical faculty at Beijing Anzhen Hospital). Briefly, plaque tissue was dried overnight and fixed in cold acetone for 10 min and washed for staining. Tissues were then incubated in primary antibody anti-CXCR4 (1:300, ab124824, Abcam Inc.) and anti-CD68 (1:200, ab955, Abcam Inc.) in adjacent sections, respectively, at 4 °C overnight and labeled with 2nd antibody-HRP and with 3.3′-diaminobenzindine (DAB) (Sigma-Aldrich) for visualization. Subsequently, tissue slides were washed in running tap water for 5 min. Finally, slides were dehydrated in alcohol with graded concentration (30% to 70% to 100%). All slides were mounted for microscope imaging.

### Statistical analysis

Patients were classified with two methods. Firstly, patients were divided into high (prevalence of cardiovascular risk factors or age ≥ 70) and low-risk groups (lacking of cardiovascular risk factors and age < 70). Unpaired Student’s t tests were used for group comparison of mean TBR_max_ values of patients as well as segmental TBR_max_ values. Secondly, we identified a mean TBR_max_ value (all arterial segments) of 1.7 based on ROC analysis (AUC = 0.602), which was the best threshold to seperate high and low risk groups. Thirdly, unpaired Student’s t tests were used to compare the prevalence of cardiovascular risk factors of patients groups above and below the mean TBR_max_ threshold value of 1.7. Additionally, paired Student’s t tests were used for group comparison of mean TBR_max_ values at baseline and follow-up scans. Statistical comparison among the different arterial lesions were performed using one-way ANOVA, with post-hoc Games Howell tests which were performed to confirm where the significant differences occurred among different arterial segments. Mann-Whitney U rank sum tests were used for non-parametric data. The Spearman correlation coefficient were used to assess the correlations between mean TBR_max_ and age, BMI as well as the occurrence of cardiovascular risk factors.

Linear relations regression between TBR_max_ at baseline and follow-up were assessed using Pearson’s regression analysis. In addition, Bland–Altman plots with limits of agreement were used to assess the agreement between baseline and follow-up measurements. Each scan of all patients was analyzed twice within 3 weeks by one experienced reader, and intra-observer agreement was assessed with a 1-way random effects model with absolute agreement. Additionally, inter-observer agreement was also assessed with a 2-way mixed effects model with absolute agreement by a second experienced reader. Intraclass correlation coefficients (ICCs) with 95% confidence intervals were calculated to test inter-observer and intra-observer agreement for TBR. We used a two-way random ICCs greater than 0.8 as an indicator for excellent reproducibility. All statistical analysis was performed in SPSS v. 19 (SPSS Inc., Chicago, IL). *P*-values <0.05 were considered statistically significant.

## Results

### [^68^Ga]Pentixafor arterial uptake and expression of CXCR4 within inflamed plaques

**Fig. 1:**
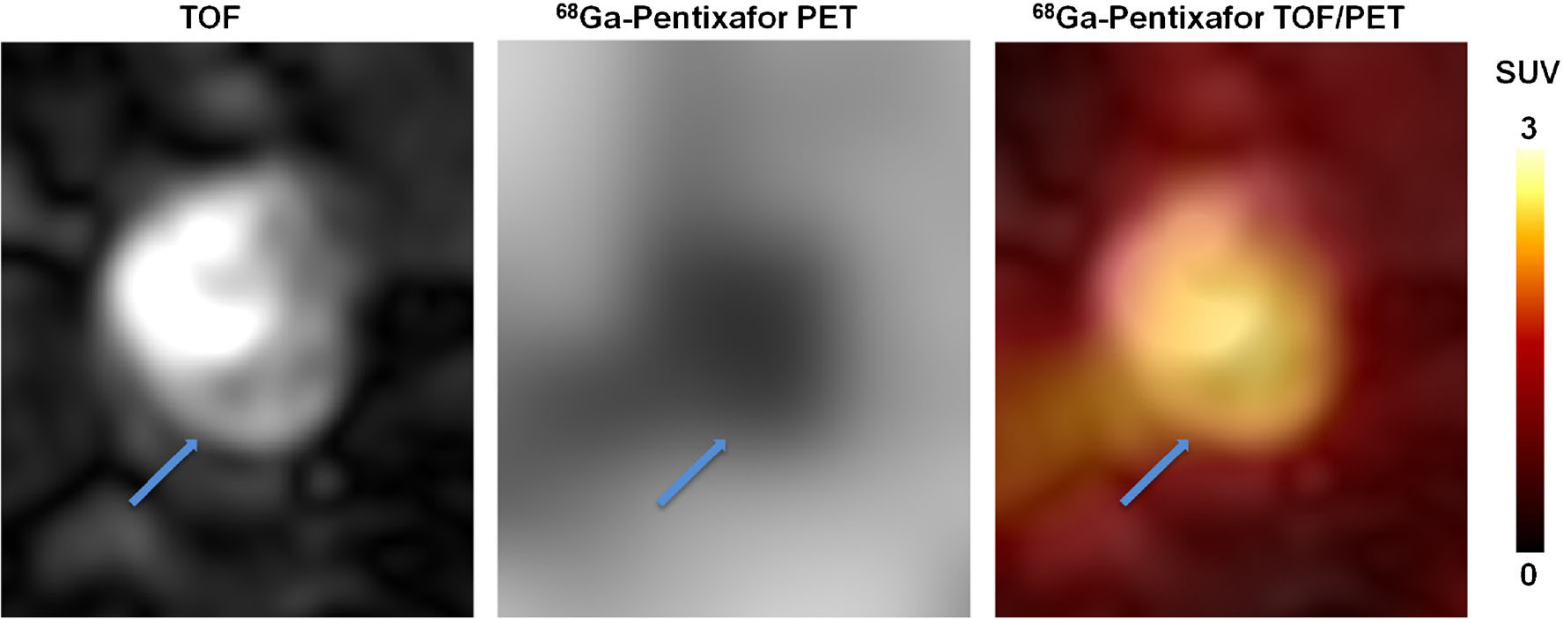
Representative axial slices of [^68^Ga]Pentixafor TOF-PET of the right carotid artery in a 71-year-old male lymphoma patient. Increased focal [^68^Ga]Pentixafor Uptake was detected at a carotid artery stenosis (arrow)

**Fig. 2:**
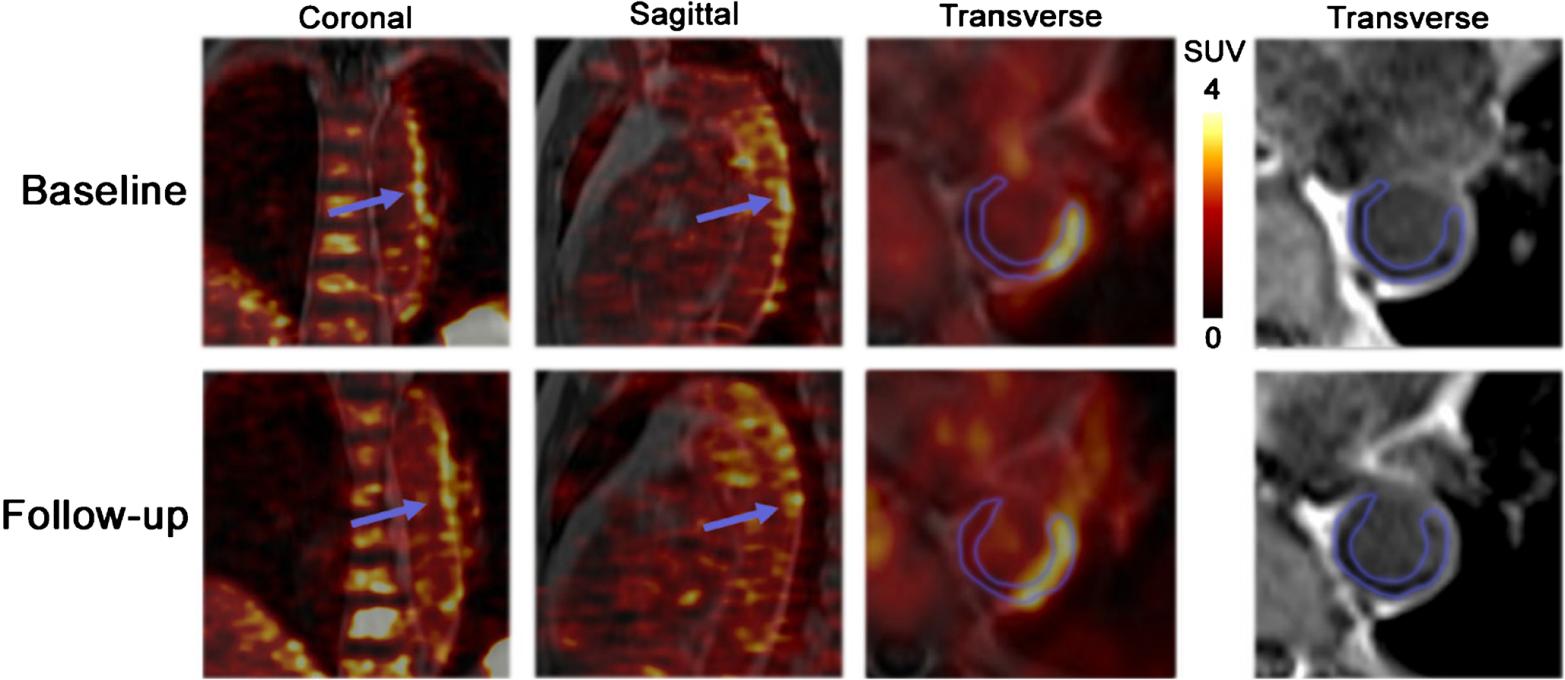
Reproducibility of [^68^Ga]Pentixafor PET imaging in atherosclerotic plaques from a representative scan of a 76-year-old female lymphoma patient. Co-localized uptake in both baseline and follow-up scans was observed (time interval = four months). Transverse view of PET/MRI scan showed spatial accumulation of [^68^Ga]Pentixafor within the mixed atherosclerotic lesions in the descending aortas

**Fig. 3:**
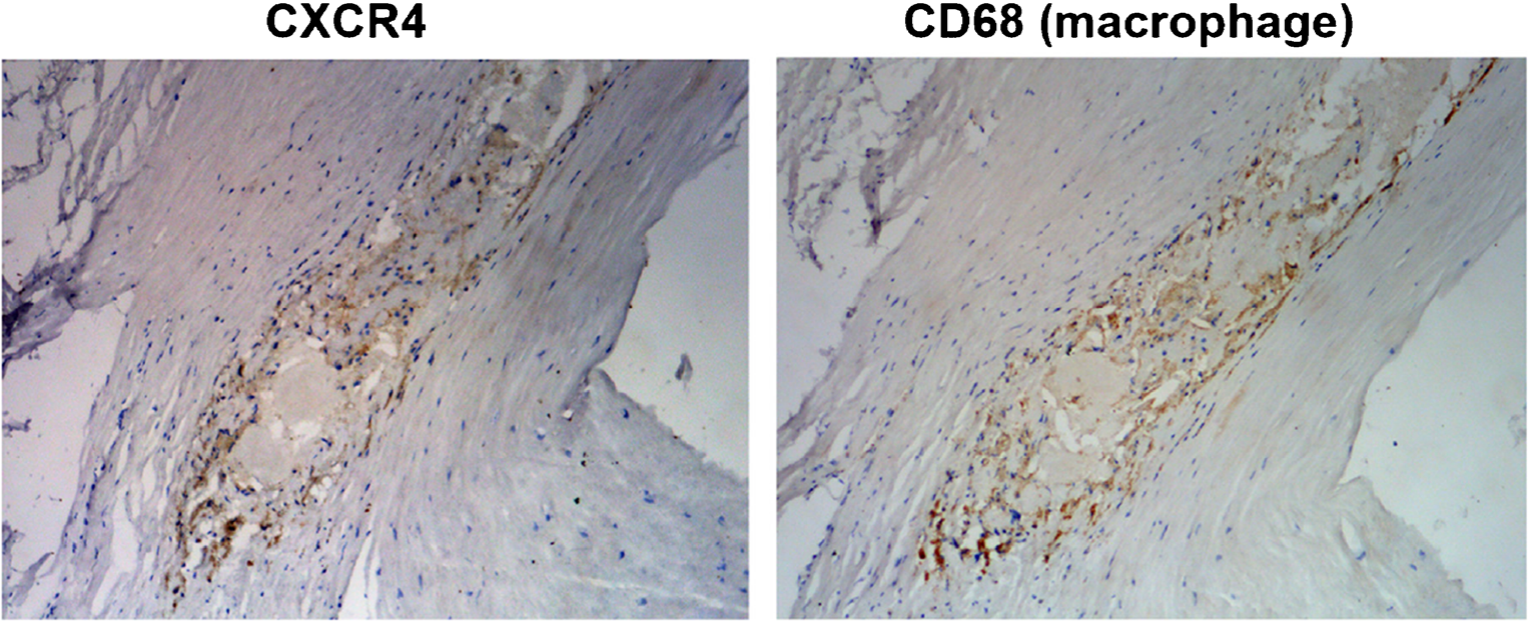
CXCR4 expression in a representative inflamed carotid plaque lesion. Brightfield micrographs showed brown chemoimmunoreactive CXCR4 and CD68 (macrophage) staining. Co-localized CXCR4 and CD68 expression was observed in these two adjacent sections

At baseline, focal [^68^Ga]Pentixafor arterial uptake was observed at 611 sites in 34 of 38 patients. Among all assessed arteries, the descending aorta was the vessel segment with the highest plaque counts (*n* = 225), followed by the abdominal aorta (*n* = 168) the aortic arch (*n* = 83), the common carotid arteries (*n* = 74), and the ascending aorta (*n* = 61) (Table 2). In addition, the mean uptake ratio (TBR_max_) of [^68^Ga]Pentixafor was significantly higher in the descending aorta (1.9 ± 0.4) and the abdominal aorta (1.9 ± 0.4) (*p* < 0.05) in comparison with uptake ratios within other arterial segments (Table 2). Figure 1 illustrates a representative [^68^Ga]Pentixafor focal uptake of a carotid arterial stenosis, Fig. 2 shows a reproducible [^68^Ga]Pentixafor uptake along the atherosclerotic descending aorta at baseline and follow-up scans. CXCR4 protein expression was co-localized with macrophage infiltration in atherosclerotic plaques (Fig. 3).

**Table 2:** Distribution of [^68^Ga]Pentixafor uptake in arterial lesions at baseline

	Carotid arterial lesions	Aortic arch lesions	Ascending aortic lesions	Descending aortic lesions	Abdominal aortic lesions	All lesions
Number of lesions	74	83	61	225	168	611
Mean TBR_max_ (Mean ± SD)	1.7 ± 0.3	1.8 ± 0.2	1.7 ± 0.2	1.9 ± 0.4	1.9 ± 0.4	1.8 ± 0.4

### Relation of CXCR4 expression with cardiovascular risk factors

**Fig. 4:**
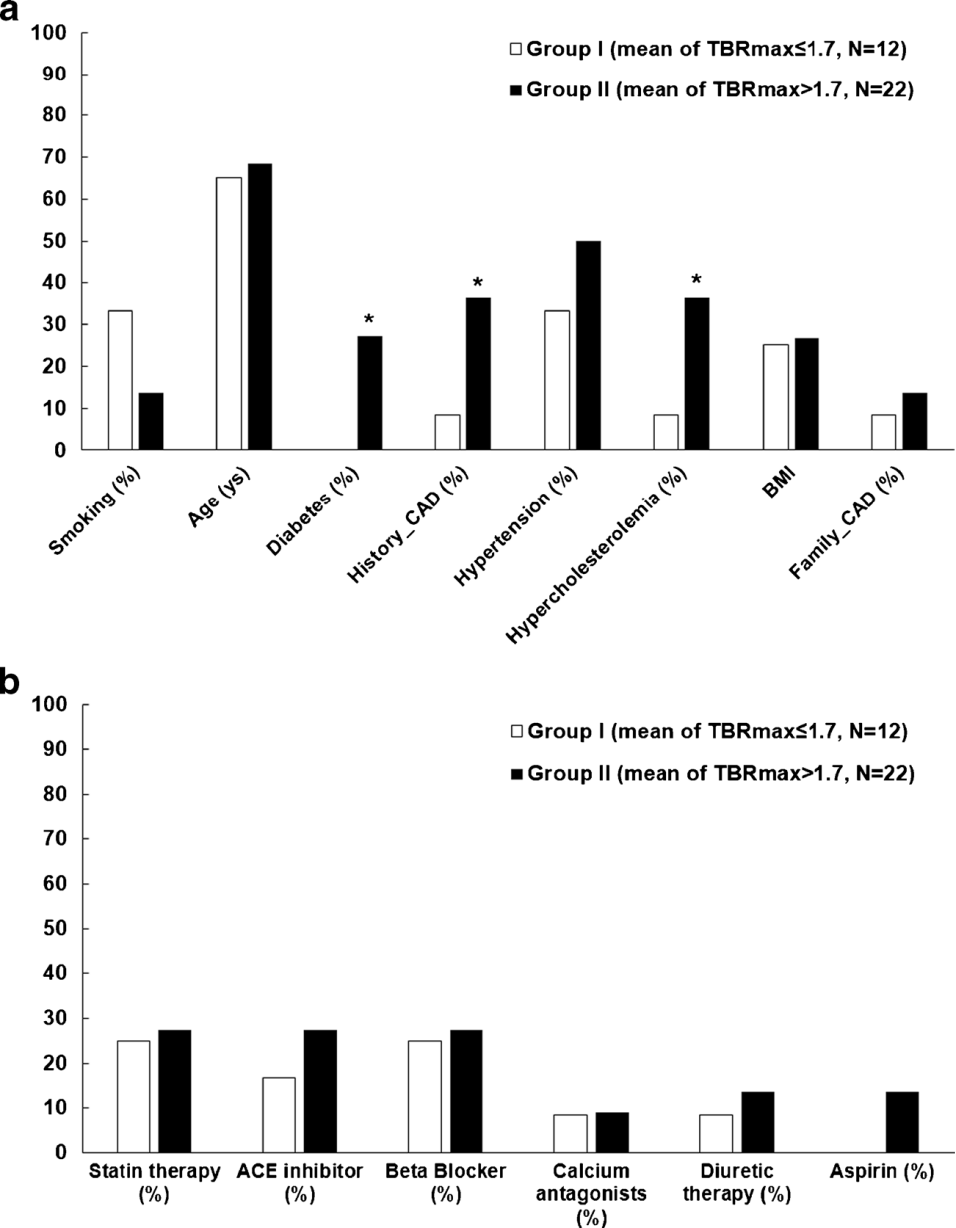
**a** Incidence of major cardiovascular risk factors and **b** Medications proportion in according to a mean TBRmax cut-off value of 1.7. CAD: cardiovascular diseases, BMI: body-mass index. ACE: Angiotensin-converting-enzyme

There was a significant correlation between sex and uptake ratios (men: 1.9 ± 0.3 vs women: 1.7 ± 0.2; Spearman correlation coefficient *r* = 0.4; *p* < 0.05). With regard to cardiovascular risk factors, patients with mean TBR_max_ > 1.7 showed a significantly higher incidence of diabetes (27.3% vs 0%, p < 0.05), hypercholesterolemia (36.4% vs 8.3%, p < 0.05) and history of cardiovascular disease (36.4% vs 8.3%, p < 0.05) compared to those with mean TBR_max_ ≤ 1.7 (Fig. 4). The overall mean TBRmax from high-risk patients (*n* = 19) were significantly higher than from low-risk patients (*n* = 15) (1.9 ± 0.3 vs 1.7 ± 0.2, p < 0.05) (Table 3).

**Table 3:** Comparison of [^68^Ga]Pentixafor mean TBR_max_ values between low-risk patients and high-risk patients

		Carotid arterial lesions	Aortic arch lesions	Ascending aortic lesions	Descending aortic lesions	Abdominal aortic lesions	**All lesions**
Low Risk Group	MeanTBR_max_ (Mean ± SD)	1.3 ± 0.3	1.6 ± 0.4	1.5 ± 0.3	1.7 ± 0.2	1.8 ± 0.3	**1.7 ± 0.2**
High Risk Group	meanTBR_max_ (Mean ± SD)	1.5 ± 0.4	1.8 ± 0.3	1.5 ± 0.4	2.0 ± 0.4	2.0 ± 0.4	**1.9 ± 0.3**
P value		0.12	<0.05	0.93	<0.05	0.25	**<0.05**

P values were calculated using unpaired Student’s t-test to compare the mean TBR_max_ values.

### Reproducibility of arterial [^68^Ga]Pentixafor uptake

**Fig. 5:**
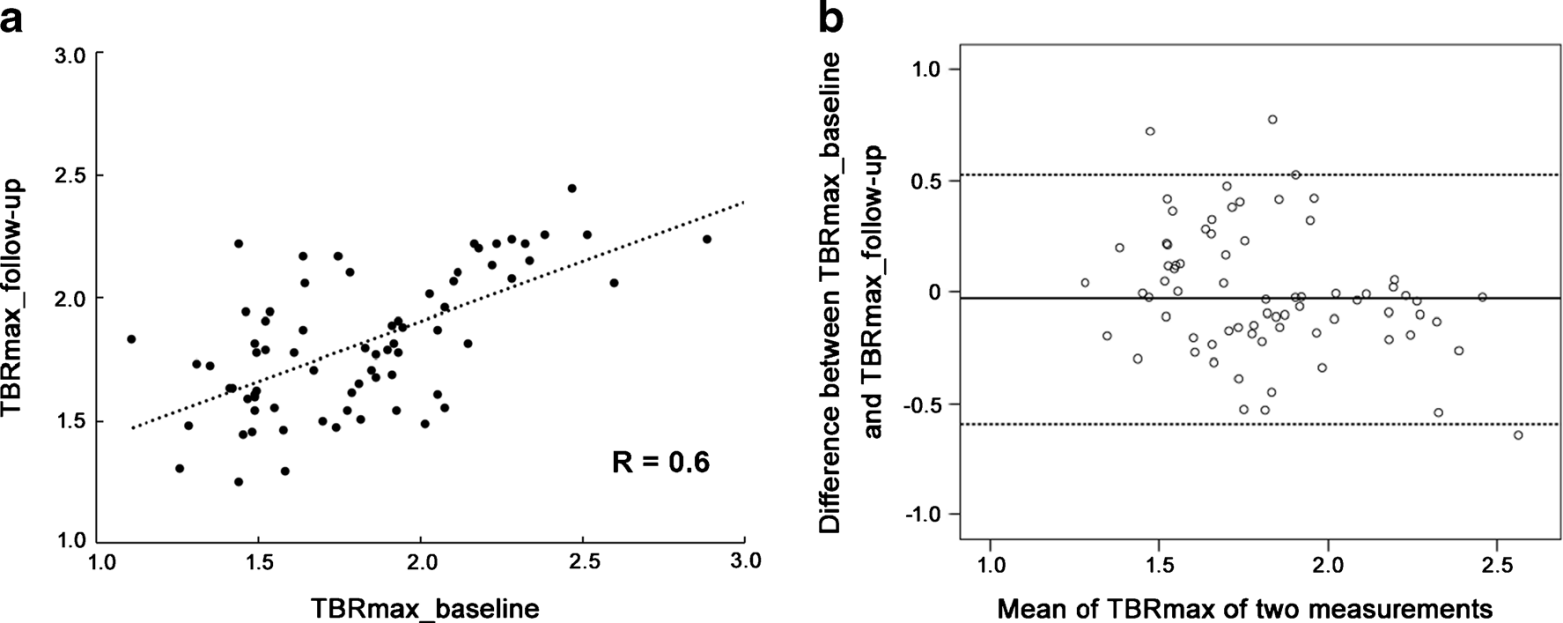
**a**, Pearson linear regression analysis (*r* = 0.6, *p* < 0.01) between TBRmax at baseline scans and TBRmax at follow-up scans. **b**, Bland Altman analysis of the agreement of maximum TBR within atherosclerotic lesions between baseline and follow-up, with a lower bias of −0.03 for the mean TBRmax

[^68^Ga]Pentixafor PET/MRI showed good reproducibility during the two examinations regarding no significant different mean of TBR_max_ value derived from corresponding lesions between two examinations (Table 4). TBR_max_ uptake values of lesions obtained from the baseline and follow-up scans showed a good correlation (*r* = 0.6, *p* < 0.01) (Fig. 5a) with a lower bias for mean TBR_max_ (−0.03), indicating an excellent agreement between the two scans (Fig. 5b). The inter- and intra-reader intraclass correlation coefficients along with 95% confidence intervals for the TBR were 0.81 and 0.9, respectively.

**Table 4:** Lesional [^68^Ga]Pentixafor uptake at baseline compared to follow-up

		Carotid arterial lesions	Aortic arch lesions	Ascending aortic lesions	Descending aortic lesions	Abdominal aortic lesions	All lesions
Number of lesions		5	11	7	25	24	72
Baseline	MeanTBR_max_ (Mean ± SD)	1.6 ± 0.2	1.8 ± 0.3	1.7 ± 0.2	1.9 ± 0.4	1.9 ± 0.4	1.8 ± 0.3
Follow-up	meanTBR_max_ (Mean ± SD)	1.5 ± 0.2	1.9 ± 0.2	1.7 ± 0.1	1.9 ± 0.3	1.8 ± 0.3	1.8 ± 0.3
*P* value		0.17	0.31	0.95	0.20	0.48	0.93

*P* values were calculated using paired sample t-tests to compare the mean TBR_max_ of baseline and follow-up scans.

## Discussion

In this present study, we quantitatively assessed [^68^Ga]Pentixafor PET/MRI in large arteries. Focal arterial uptake of [^68^Ga]Pentixafor was observed in atherosclerotic lesions, with high reproducibility determined between baseline and follow-up scans. There was a strong relation between increased uptake ratios and some representative cardiovascular risk factors.

### Biomarker of CXCR4 for atherosclerosis

The function of the CXCL12-CXCR4 axis in severe cardiac inflammatory processes, such as myocardial infarction, most likely is related to progenitor cell recruitment to the injured myocardium. CXCL12, the ligand of CXCR4, was demonstrated to be expressed by vascular smooth muscle cells (vSMCs), endothelial cells, and macrophages in atherosclerotic, but not in healthy arteries. Pioneering studies have also confirmed a potential contributory role of bone marrow-derived vascular progenitor cells in the progression of atherosclerosis [26, 27]. Nevertheless, their exact role in native atherosclerosis progression still remains largely unclear.

Various immune cell types participate in plaque growth, most of which demonstrate significant expression of CXCR4, and its regulation was also mediated by multi-factors with contentious perspectives in its functions in atherosclerosis progression. A number of reports pointed to a pro-atherosclerotic role for CXCR4. One major concept demonstrated that as alternative ligand, the macrophage migration inhibitory factor (MIF) directly binds to the vascular CXCR4 protein, and the process triggered vascular monocyte and T-cell recruitment, and subsequently induced atherosclerotic progression [2]. In functional perspective of inflammatory activity and CXCR4 association during atherosclerosis progression, the oxidized low-density lipoprotein (Ox-LDL) could have significantly induced CXCR4 expression in the macrophage [11]. A recent study reported that an enhanced vascular hypoxic process could increase intra-plaque CXCR4 expression [28], which enhanced the atherosclerotic progression significantly. However, CXCR4 expression was also proven to have a protective role in atherosclerosis, due to CXCR4 blockade-enhanced plaque initiation [29], which was associated with increased activated neutrophils in the blood and an induced neutrophil component, in accordance with atherosclerotic apoptosis and inflammation [30, 31]. Due to these controversial concepts of CXCR4 functions in atherosclerosis, a systemic study was proposed to reveal the expression of CXCR4 on different types of plaque considering the specific distribution of CXCR4 proteins [32] that could serve as an efficient biomarker for atherosclerosis imaging. Ilze Bot et al. found that, in comparison within early atherosclerotic lesions and advanced relatively stable atherosclerotic lesions, CXCR4 expression was significantly pronounced in advanced unstable lesions [32]. This finding might support the concept that CXCR4 plays a recruitment role for leukocytes into the vessel wall, but a homeostatic role for neutrophils in the blood pool. Interestingly, in our observation, arterial local CXCR4 expression and macrophage infiltration overlapped, which support the fact that intra-plaque CXCR4 protein might be a positive marker for inflamed plaques.

### Atherosclerosis PET imaging

Nowadays, numerous biomarkers for PET imaging have been validated, and macrophage imaging has been considered a major target for atherosclerosis [33]. Among these macrophage tracers, ^18^F-FDG is the most available and widely used in clinical research, with increased glucose uptake in lipid-laden macrophages. However, ^18^F-FDG plaque imaging is limited by low specificity and significant myocardial uptake.

In our study [^68^Ga]Pentixafor uptake was only significantly correlated with sex; the lack of statistical significance might be due to the small size of our sample. Nevertheless, [^68^Ga]Pentixafor PET shows a clear view of vascular uptake with a relatively clear background. Notably, in the present study, we found that patients with higher uptake of [^68^Ga]Pentiaxafor showed a higher incidence of hypertension, diabetes, hypercholesterolemia, and prior cardiovascular diseases compared with patients with lower uptake ratios. This significant quantitative relationship between increased uptake ratios and increased presence of conventional cardiovascular risk factors might suggest a potential role for [^68^Ga]Pentixaifor PET/MRI in the evaluation of diseased vessels. Remarkably, significant expression of CXCR4 protein was confirmed by immunohistology in inflamed vulnerable plaque lesions, which is co-localized with macrophage expression [9]. This evidence of CXCR4 biodistribution combined with our imaging results might support the concept of using CXCR4 as a biomarker to characterize inflammatory atherosclerosis.

### Limitations

There are several limitations to this study. Given the limited number of observations, no final conclusions can be drawn yet. Second, dedicated MRI sequences were not performed for the aortic wall assessment, so that accurate allocation and characterizations of focal tracer uptake as well as MRI lesion characteriszation, such as intra-plaque calcifications, or fibrous caps were not possible. We also could not ignore the evidence of the significant limitation of partial volume effects in small-sized atherosclerotic lesions and MRI-based attenuation correction in PET imaging, which might lead to an underestimation of tracer accumulation [13]. Furthermore, we also cannot exclude biologic processes of CXCR4 underlying cancer and the influence of anticancer therapies or the hormone response of immune-modulatory drugs. In particular, previous studies have demonstrated that the use of statin therapy for atherosclerosis could induce significant downregulation of the expression of CXCR4 on monocyte subsets [5, 34]. In a group comparison, we used an arbitrary cut-off value of TBRmax = 1.7 based on the visualization for hot lesions, which is lacking supporting evidence. Further study concerning the correlation between TBR value and histology finding are warranted.

Further assessments of the effect of statins or angiotensin-converting enzyme inhibition therapy are suggested. Despite pioneering immunohistological studies performed to reveal the biodistribution of the CXCR4 protein within vulnerable lesions; nevertheless, CXCR4 also shows a protective role in atherosclerotic arteries, and further comprehensive in vivo and in vitro assessments of dedicated [^68^Ga]Pentixaifor PET/MRI and immunohistology in distinguished types of plaques lesions and cell types are suggested.

## Conclusion

Focal [^68^Ga]Pentixafor uptake was detected in atherosclerotic lesions with high reproducibility over time, and significantly increased uptake ratios were found in patients with typical cardiovascular risk profiles. Quantification of [^68^Ga]Pentixafor PET could serve as an in vivo imaging tool for the identification of inflammatory atherosclerosis and, together with dedicated MRI, might provide new insights into the pathobiology of atherosclerosis.
